# Dengue as a cause of acute undifferentiated fever in Vietnam

**DOI:** 10.1186/1471-2334-6-123

**Published:** 2006-07-25

**Authors:** Hoang Lan Phuong, Peter J de Vries, Tran TT Nga, Phan T Giao, Le Q Hung, Tran Q Binh, Nguyen V Nam, Nico Nagelkerke, Piet A Kager

**Affiliations:** 1 Division of Infectious Diseases, Tropical Medicine and AIDS, Academic Medical Center, P.O. Box 22700, 1100 DE Amsterdam, the Netherlands Amsterdam, The Netherlands; 2 Department of Tropical Diseases, Cho Ray Hospital, 201 B Nguyen Chi Thanh, Ho Chi Minh City, Vietnam; 3 Department of Microbiology, Cho Ray Hospital, 201 B Nguyen Chi Thanh, Ho Chi Minh City, Vietnam; 4 Binh Thuan Malaria and Goiter Control Center, 133A Hai Thuong Lan Ong, Phan Thiet, Vietnam; 5 Dept of Community Medicine, United Arab Emirates University, P.O. Box 17666 Al Ain, United Arab Emirates

## Abstract

**Background:**

Dengue is a common cause of fever in the tropics but its contribution to the total burden of febrile illnesses that is presented to primary health facilities in endemic regions such as Vietnam, is largely unknown. We aimed to report the frequency of dengue as a cause of fever in Binh Thuan Province, to describe the characteristics of dengue patients, and analyze the diagnostic accuracy of the health care workers and the determinants of the diagnostic process.

**Methods:**

All patients presenting with acute undifferentiated fever at twelve community health posts and one clinic at the provincial malaria station, Binh Thuan Province, a dengue endemic province in southern Vietnam, were included. Record forms were used to fill in patient and diseases characteristics, pre-referral treatment, signs and symptoms, provisional diagnosis and prescribed treatment, referral and final outcome. Serum samples were collected at first presentation and after 3 weeks for serologic diagnosis.

**Results:**

2096 patients were included from April 2001 to March 2002. All 697 patients with paired serum samples were tested for dengue virus IgM and IgG. Acute dengue was found in 33.6% cases and past dengue virus infections were found in 57.1% cases. Acute primary infections were more common among children under 15 years old than among adults (7.7% vs. 3.5%, p value < 0.001). Younger age significantly predicted acute dengue (RR per increasing year of age (95 % CI): 0.986 (0.975–0.997, p value = 0.014). 48.9% of cases with clinical diagnosis of acute dengue were serologically confirmed and 32.5% of cases without clinical diagnosis of acute dengue were positive by serology after all (OR = 1.981, p value 0.025, 95% CI: 1.079 – 3.635). Tourniquet test was not a predictor for dengue diagnosis.

**Conclusion:**

Dengue is responsible for one third of the fevers presented to the public primary health services in Binh Thuan, southern Vietnam. It presents as a highly unspecific illness and is hardly recognized as a clinical entity by primary physicians.

## Background

Communicable diseases constitute a substantial part of the health problems in Vietnam. Several disease control programs are in place to control infectious diseases. In Binh Thuan province, southern Vietnam, the total burden of infectious diseases presented to the public health services is not known. The only data available were collected by district and provincial hospitals and these data do not reflect the true incidence of the respective diseases. Moreover, most febrile diseases are usually not specified as to their cause and treatment is rather generic, typically with antipyretics and antibiotics [1]. Although seemingly pragmatic, polypharmacy also leads to unnecessary adverse drug events, increased costs and selection of resistant microorganisms.

National data show that dengue is common in Vietnam, but despite the National Dengue Control Program, which was launched in 1998, notification of dengue is probably incomplete. In 2001, dengue control was still in its infancy and was mainly based on passive case detection and notification of complicated cases of dengue hemorrhagic fever and dengue shock syndrome. Uncomplicated dengue was not recorded, and it was unknown if this was recognized as such.

In 2001 we started a study into the causes of fever presented to public facilities for primary health care (in the following to be called "health posts") in Binh Thuan province. In a previous study we analyzed the diagnostic process of health care workers and concluded that this was very unspecific, did not distinguish viral from bacterial infections and led to polypharmacy and very frequent prescription of antibiotics. Here we report on the frequency of dengue, confirmed by serology, as a cause of fever in Binh Thuan Province, describe the characteristics of dengue patients, and analyze the diagnostic accuracy of the health care workers' presumptive diagnosis and determinants of the diagnostic process.

## Methods

### Study site and population

**Figure 1:**
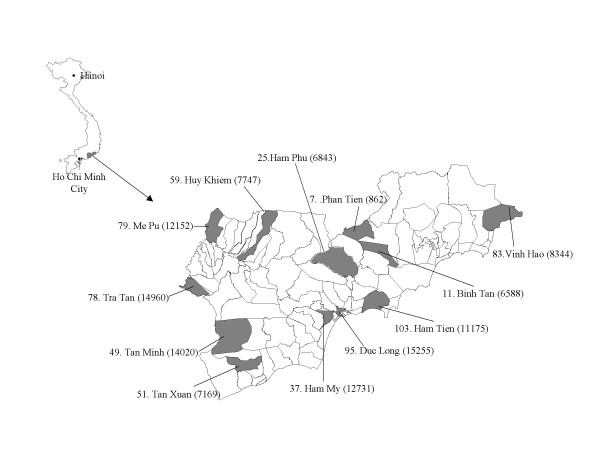
Administrative map of Binh Thuan indicating the communes (name and number) participating in the study. Between brackets the population in 2000.

The study started in April 2001 in Binh Thuan province (Figure 1) in southern Vietnam. The study area has been described previously [1]. In brief, Binh Thuan has a population of approximately 1.1 million on an area of 7992 km^2^, divided over 115 administrative communes. (Source: Statistical Yearbook 2001 – Binh Thuan Statistics Office, Phan Thiet). A decade ago, Binh Thuan was a relatively poor region, but the provincial annual income per capita increased rapidly from US$118 in 1990 to US$278 in urban areas and US$230 in rural areas in 2000 (the national income per capita was at time US$374).

Health care in Vietnam is structured at four levels: national policy and steering bodies, provincial health services, district health centers and commune-level health centers (health posts). Health posts provide curative care, antenatal care, obstetric and mother and child health care, implement prevention programs such as immunization, health promotion and vector control and serve as the gateway for the national diseases control programs.

In Binh Thuan province all health posts in the malaria endemic zones offer microscopic diagnosis and treatment of malaria; the other zones are not completely covered by microscope posts [2]. For additional examination and treatment of severe diseases, patients are referred to the district health centers. These are typically small hospitals that offer basic diagnosis and treatment, have a small laboratory for basic biochemistry and blood counting, an ultrasound or X-ray facility and basic operating theatre. There is one provincial hospital offering a rather complete range of general care, including basic microbiological laboratory facilities. For specialized and intensive care, patients are usually referred to one of the specialized hospitals in Ho Chi Minh City at a distance of 150 km south of Binh Thuan province.

The private health sector increased during the last decade of "open policy" and relaxation of the government's control. There is no good estimate of the number of patients attending private clinics and dispensaries. Private clinics are virtually absent in the poorest regions where the population consists of ethnic minorities. In these regions malaria is one of the most important diseases in the differential diagnosis of fever and malaria is exclusively handled by the public health services. In the (semi-)urban regions and rural non-malarious regions, the impact of the private sector is probably much greater.

For this study we selected twelve, not adjacent communities that represented rural and (semi-) urban, lowland and highland communes, and the province's ethnic population structure (Figure 1). In addition, the clinic of the provincial malaria station in Phan Thiet, the capital, where suspected malaria patients come for diagnosis and treatment, also participated.

The medical staffs of the participating health posts included assistant doctors – who received three years of training at a medical school-, and fully qualified, university trained, doctors (MD).

All patients presenting with acute undifferentiated fever (AUF) were included in this study. AUF was defined as any febrile illness of duration less than 14 days, confirmed by an axillary temperature ≥ 38.0°C, without indication of either severe systemic or organ specific disease and not being malaria, which was excluded by microscopic examination of a thick blood smear.

### Data collection

After informed consent, record forms were filled in for all AUF patients recording identifiers (age, sex, occupation, and address), recent exposure factors (contact with fresh water or flooded terrains, work in paddy fields, visit to forest or work in forest) and characteristics of disease (duration, symptoms and signs, including the results of the tourniquet test), self-treatment, presumptive diagnosis and prescribed treatment, referral and final outcome. Presumptive diagnoses such as "acute fever" and "viral infection" were reclassified to "undifferentiated fever".

Data were entered by the attending healthcare worker immediately upon presentation of the patient. All health posts were monitored at monthly visits by the research team from Cho Ray Hospital, Ho Chi Minh City.

Serum samples were collected for sero-diagnosis. An "acute" sample was collected at first presentation (t0) and all included patients were asked to come back after approximately 3 weeks for re-assessment and collection of a second, "convalescence", serum sample (t3). Serum samples were stored at -20°C at the study sites until monthly transfer to Cho Ray hospital, where they were stored at -70°C.

### Serological confirmation of dengue virus infection

All complete pairs of acute and convalescence serum samples were tested for dengue with IgG and IgM-Capture ELISA (Focus Technologies Inc., Cypress, CA, USA). This commercially available assay has been evaluated and was found to be sufficiently sensitive and specific for the sero-diagnosis in clinical serum samples [3]. The tests were performed as described previously [4]. In brief, a four fold increase of antibody concentrations between t0 and t3 was considered significant. The IgM concentration on t3, relative to the IgG concentration on t3 was also used as a criterion. Acute primary dengue virus infection was defined as positive IgM on t3 with an IgM/IgG ratio on t3 greater than one. A positive IgM on t3 with an IgM/IgG ratio on t3 less than one, or a negative IgM reaction on t3 but with a positive IgG t3 and a fourfold molar increase of IgG between t0 to t3 were classified as acute secondary dengue. A negative IgM reaction on t3, a positive IgG on t3 but without a fourfold increase between t0 and t3 was classified as "not acute dengue but past infection", and a case of both negative IgM and IgG on t3 was classified as "no dengue".

### Ethical considerations

The study was approved by the Review Board of the Cho Ray Hospital, Ho Chi Minh City, Vietnam. The study was explained and discussed in meetings with provincial authorities and staff of the health posts. All patients, (or, for children, the parents or guardian) gave their written informed consent.

### Statistics

Statistical analysis was done using SPSS (version 12.02, SPSS Inc. Illinois). The Chi-square test was applied to compare frequencies of categorical data. Odds ratios were calculated as an estimate of diagnostic accuracy. Binary (logistic) regression was used to compare the disease characteristics of acute dengue to those of other fevers.

## Results

From April 2001 to April 2002, 2108 patients were enrolled in this study. After exclusion of 12 violations of inclusion criteria (axillary temperature below 38° (2) or not recorded (3), signs of organ specific disease such as urinary tract infection (2), mastitis (1), otitis media (1), cholangitis (2), gingivitis (1)), 2096 cases remained for analysis. Serological analysis was performed on 697 cases of whom acute and convalescent serum samples were available.

The characteristics of the total study population and patients of whom two sera were available are shown in Table 1.

**Table 1:** Study population

	Population without paired serum samples (%)	Population with paired serum samples (%)	P-value
Total number	1399	697	
Females/males (ratio)	611/788 (0.78)	256/441 (0.58)	0.002
Age*(years, median, range)	15 (1–82)	24 (4 – 82)	<0.001
≤ 15 year	715 (51)	181 (26)	
> 15 years	682(49)	516 (74)	
**Occupation**			<0.001
Farmer	473 (33.8)	347 (49.8)	
Pupil	568 (40.6)	200 (28.7)	
Child at preschool age	159 (11.4)	4 (0.6)	
Worker or fisherman	109 (7.8)	67 (9.6)	
Retired age	30 (2.1)	11 (1.6)	
Government employee	21 (1.5)	17 (2.4)	
Other	39 (2.8)	51 (7.3)	
**Presumptive diagnosis**			<0.001
Undifferentiated fever	689 (49.2)	387 (55.5)	
Pharyngitis	361 (25.8)	140 (20.1)	
Dengue fever	135 (9.6)	45 (6.5)	
Tonsillitis	118 (8.4)	29 (4.2)	
Typhoid fever	39 (2.8)	36(5.2)	
Diarrhoea/Enteritis	31 (2.2)	27 (3.9)	
Leptospirosis	2 (0.1)	9 (1.3)	
Hepatitis	3 (0.2)	7 (1.0)	
Acute respiratory tract infection	1 (0.1)	1 (0.1)	
Other**	20 (1.4)	16 (2.3)	

* 2 cases missing age

**Other: Allergy, arthritis, clinical malaria, gastritis, lymphadenitis, measles, mumps, sinusitis, varicella.

### Dengue serological diagnosis

All paired serum samples were tested for dengue virus IgM and IgG. Acute dengue was found in 234 (33.6%) cases, including 32 (4.6%) cases of acute primary dengue and 202 (29.0%) cases of acute secondary dengue. Signs of past dengue virus infection were found in 398 (57.1%) patients. The distributions of serological diagnoses among children under 15 years old versus adults were respectively: acute primary dengue 7.7% vs. 3.5%; no dengue 6.4% vs. 17.7%; acute secondary dengue 27.6% vs. 29.5%; status past dengue 47.0% vs. 60.7% (overall, Chi-square 28.117 (df = 3); p value < 0.001).

### Characteristics of patients with acute dengue

Characteristics of patients with acute primary or secondary dengue or another febrile disease are shown in Table 2. This table omitted some factor and characteristic that appeared in small frequency and were not different among 3 groups (Exposure factors: worked in paddy fields, recently visited forest, helped in clearing forest and recent contact with fresh water; Symptoms of 3 groups – Other febrile disease, Acute primary dengue and Acute secondary dengue, respectively (%): Constipation (4.3;0;1.5), Rash (4.3;0;4.0), spontaneous bleeding (0.4;0;3.0), and altered consciousness (0.2;0;0); Findings at physical examination (%): jaundice (4.8;3.1;2.0), rash (3.0;0;1.0), bruises (2.8;0;1.5), lymphadenitis (2.6;0;1.5), lymphadenopathy (2.2;0;1.0), hepatomegaly (1.3;0;1.0), splenomegaly (0.9;0;0.5), eschar (0.6;0;0), Vesicles (0.4;0;0.5)).

**Table 2:** Exposure, signs and symptoms of febrile patients, presenting to a primary health care facility, with serologically confirmed acute primary dengue and secondary dengue and febrile patients without dengue.

	**Other febrile disease ^a ^** **(n = 463)**	**Acute primary dengue ^b ^** **(n = 32)**	**Acute secondary dengue ^c ^** **(n = 202)**	**P value**
**Age **(median, range)	24 (4 – 82)	19 (5 – 68)	23 (5 – 74)	0.013*
**Sex, no. (%)**				ns
Male	288 (62.2)	19 (59.4)	134 (66.3)	
Female	175 (37.8)	13 (40.6)	68 (33.7)	
**Exposure factors, no. (%)**				
Waded through flooded terrain	61 (13.2)	4 (12.5)	34 (16.8)	0.003 ^c^
Days of illness (median, range)	2 (0 – 10)	3 (1 – 10)	2 (0 – 11)	ns
Days of fever (median, range)	1 (0 – 9)	1 (0 – 10)	2 (0 – 11)	ns
Days in bed (median, range)	1 (0 – 8)	1 (0 – 7)	2 (0 – 9)	ns
**Symptoms, no. (%)**				
Headache	446 (96.3)	27 (84.4)	198 (98.0)	0.012 ^b^
Anorexia	387 (83.6)	24 (75.0)	161 (79.7)	ns
Myalgia	272 (58.7)	15 (46.9)	126 (62.4)	0.047 ^c^
Sore throat	196 (42.3)	17 (53.1)	81 (40.1)	ns
Backache	160 (34.6)	10 (31.3)	61 (30.2)	ns
Nausea	151 (32.6)	8 (25.0)	53 (26.2)	ns
Cough	133 (28.7)	17 (53.1)	62 (30.7)	ns
Running nose	118 (25.5)	13 (40.6)	31 (15.3)	0.009 ^c^
Arthralgia	67 (14.5)	5 (15.6)	25 (12.4)	0.038 ^b^
Abdominal pain	66 (14.3)	7 (21.9)	33 (16.3)	0.005 ^c^
Vomitus	63 (13.6)	5 (15.6)	24 (11.9)	ns
Diarrhoea	35 (7.6)	1 (3.1)	9 (4.5)	0.047 ^c^
**Findings at physical examination, no. (%)**				
Tender muscles on palpation	241 (52.1)	16 (50.0)	87 (43.1)	ns
Red pharynx	231 (49.9)	21 (65.6)	93 (46.0)	ns
Pallor	140 (30.2)	14 (43.8)	46 (22.8)	0.047 ^b^
Rhinitis	104 (22.5)	14 (43.8)	31 (15.3)	ns
Abdominal tenderness	88 (19.0)	8 (25.0)	31 (15.3)	ns
Arthritis	67 (14.5)	5 (15.6)	25 (12.4)	ns
Dehydration	56 (12.1)	3 (9.4)	20 (9.9)	ns
Tender liver	33 (7.1)	0	11 (5.4)	ns
Conjunctivitis	27 (5.8)	3 (9.4)	13 (6.4)	ns
Tourniquet test (number of petechie/square inch >0; median; range)	n = 38 5.5 (2 – 70)	n = 1 2 (2)	n = 22 8 (1 – 55)	ns

**P value **of multinomial logistic regression; (a): reference category for (b) and (c); ns: no significant

* Kruskal Wallis Test

Binary (logistic) regression was applied to compare cases with acute (primary or secondary) dengue to other cases of fever. Overall, younger age significantly predicted for acute dengue (RR per increasing year of age (95 % CI): 0.986 (0.975–0.997, p value = 0.014). The multivariable analysis was split for children under 15 years and adults (older than 15 years). All signs and symptoms, including age, were entered into this multivariate regression model. Among adults, age was still a significant predictor in the multivariate model. Cough, abdominal pain and myalgia predicted for dengue but when these variables were tested individually with the Chi Square test, their effect was not significant. Among children some factors had a small significant predictive effect in the multivariate model, but only the finding of conjunctivitis and nausea were significantly associated with dengue (1.7% among non-dengue and 7.8% among dengue, p = 0.054 and 41.0% and 21.9%, p = 0.007, respectively).

Logistic regression analysis was also used to detect differences between acute primary and acute secondary dengue. There was no sign or symptom significantly associated with one of these two outcomes.

After consultation 37 of the 697 patients (5.3%) were admitted to the health posts, 9 with acute secondary dengue and 28 with other febrile diseases. The duration of admission was the same for the 2 groups: 5 days, range 1 to 7 days).

### Clinical diagnosis and serological confirmation of dengue

The clinical presumptive diagnosis and serological confirmation of acute dengue are shown in Table 3. The presumptive diagnosis was rather imprecise, with "undifferentiated fever" accounting for more than half of the diagnoses. We used the odds ratios of being serologically classified as acute dengue or not (i.e. another febrile disease) as an estimate of diagnostic accuracy. For example, an odds ratio of 1 implies that the presumptive diagnosis has no predictive value for the serological diagnosis of dengue. Ideally, a presumptive diagnosis of dengue would have a high predictive value for a positive dengue serum test. The comparison of the odds ratios per presumptive diagnosis illustrates the accuracy of the presumptive diagnosis.

**Table 3:** Presumptive diagnosis and serological evidence of acute dengue

Presumptive clinical diagnosis	Number of cases	Serological evidence of acute dengue			P value
			
		No positive (%)	OR*	95% CI	
*Total*	***697***	***234 (33.6)***			
Dengue fever	45	22 (48.9)	1,985	1,082 – 3,643	**0,027**
Other	652	212 (30.4)			
Undifferentiated fever	387	137 (35.4)	1,203	0,876 – 1,654	0,254
Pharyngitis	140	44 (31.4)	0,885	0,595 – 1,317	0,548
Typhoid fever	36	12 (33.3)	0,989	0,485 – 2,014	0,975
Tonsillitis	29	7 (24.1)	0,618	0,260 – 1,469	0,272
Diarrhoea	27	6 (22.2)	0,554	0,220 – 1,392	0,203
Leptospirosis	9	1 (11.1)	0,244	0,030 – 1,963	0,151
Hepatitis	7	3 (42.9)	1,490	0,331 – 6,714	0,601
Acute respiratory tract infection	1	0 (0)			
Others	16	2 (12.5)	0,276	0,062 – 1,227	0,071

* OR: odds ratio in logistic regression mode = (number of acute dengue/number of no acute dengue, among presumptive diagnosis)/(number of acute dengue/number of no acute dengue, among all other diagnoses)

Of the 45 cases that were clinically diagnosed as acute dengue, 22 were serologically confirmed (48.9%). Of the 652 cases diagnosed as not acute dengue, 212 (32.5%) were still positive by serology (OR = 1.981, p value 0.025, 95% CI: 1.079 – 3.635). The overall agreement between clinical and serological diagnosis of dengue was poor (Cohen's kappa 0.055, p value 0,024).

It was only the presumptive diagnosis "dengue" that predicted a positive serum diagnosis in a significant way. All other presumptive diagnoses did not predict a negative dengue serum test, and thus did not exclude dengue.

### Effects on diagnostic accuracy

Characteristics of health care workers who participated in the study were analyzed to their effect on the diagnostic precision. There were 43 healthcare workers of 13 study sites contributing to this study, 17 females and 26 males (female/male 1/1.5), with a mean (range) age of 36.7 years (26–56 years) and a mean (range) duration of working experience of 12.7 years (1 – 35 years). Twenty (46.5%) persons were licensed as assistant doctor and 21 (48.8%) were medical doctor (MD) including 12 former assistant doctors who were recently upgraded after additional postgraduate training.

General Log linear Analysis was used to explore the effects of gender, age, professional experience and educational level on the diagnostic accuracy (i.e. on the odds ratio of the dengue sero-diagnosis per presumptive diagnosis). There was no significant effect of any of the characteristics of the doctors.

## Discussions

This study shows that dengue is responsible for approximately one-third of all cases of fever in Binh Thuan province that present to the government operated primary out patient clinics. It has been known since long that dengue is a common cause of hospitalization in Southeast Asia and that, with epidemic oscillations; dengue significantly contributes to al causes of fever in non-hospitalized children [5-8]. The fact that dengue is also responsible for such a large proportion of febrile episodes among the entire population of an endemic area such as Binh Thuan, was underexposed. The total incidence of acute, notably primary, dengue is probably even underestimated in this study because it appeared difficult to obtain paired sera from infants and young children, the groups with the highest incidence of acute dengue [6].

The true incidence rate in Binh Thuan province cannot be estimated from this study but in a recent sero-prevalence study we showed that the IgG sero-prevalence among schoolchildren increased by age until 88% at the age of 14 years, corresponding to an 11.7% annual incidence of primary dengue [9]. The IgG- sero-prevalence in convalescent sera in this study, 86.1% (74.6% for children under 16 and 90.2 % for adults), is in the same range and is also comparable to the 79% sero-prevalence that was found among Laotian febrile patients [10].

High incidence rates of dengue do not necessarily lead to high complication rates. Dengue hemorrhagic fever and dengue shock syndrome are mainly associated with repeated infections [11,12]. Already four decades ago in Thailand it was shown that uncomplicated secondary and primary infections cannot be distinguished on clinical grounds. In highly endemic areas such as Binh Thuan, the incidence of complicated dengue is probably the tip of the iceberg which surfaces in a cyclic, epidemic, pattern, following the introduction of new dengue virus serotypes. Previous studies by Institute Pasteur, Ho Chi Minh City, demonstrated the cyclic presence of all four dengue virus serotypes in Binh Thuan province (unpublished observation). The introduction of new dengue virus types, in conditions of continuously high transmission, may be responsible for epidemics of dengue hemorrhagic fever in southern Vietnam, such as the one in 1998, which affected 438.98 cases/100,000 populations and caused 342 deaths (1.26 per 100,000 populations) [13].

Despite being so common, dengue was hardly recognized as such. In only 6.5% of cases (45/697), dengue was recorded as presumptive diagnosis and in less than half of these cases this was correct. Although the odds ratio of serological evidence for dengue was higher than for the other clinical diagnoses, the overall clinical diagnosis can be regarded as highly imprecise, and, with respect to dengue, also inaccurate. This was not caused by lacunar knowledge of the physicians but by the mere absence of discriminating signs and symptoms reported by the patients or found at physical examination. One plausible explanation for this is that patients in Binh Thuan Province present rather early in their febrile episode, mostly within two days, when symptoms are still non-specific [1]. The public primary care services in Binh Thuan Province focus on early detection of malaria and delay in seeking care is usually very short [14]. On the other hand, studies in other regions also confirmed that in out patients as well as in hospitalized patients; it is difficult to make a clinical distinction between acute dengue and other causes of fever. Similar to previous studies we frequently observed signs of pharyngitis in patients with dengue and this frustrates the differentiation from respiratory tract infections [5,15,16]. The tourniquet test is also not very helpful in making a clinical diagnosis. It is an old test for demonstrating vasculopathy or coagulopathy and it has almost completely been replaced by other laboratory tests of bleeding disorders. To date, dengue is the only disease for which the tourniquet test is still used, but in principle only for detecting dengue hemorrhagic fever. For this however, it is not very sensitive, often being negative in the presence of other signs of hemorrhagic diathesis in hospitalized children with dengue [17]. In contrast, in a previous study we showed that physicians intuitively also use the tourniquet test to diagnose uncomplicated dengue, by applying an inappropriately low threshold of the petechiae count [1]. It is questionable if the tourniquet test should still be maintained as a criterion in the diagnostic workup of dengue.

Since the clinical diagnosis of dengue fever has such a low predictive value, laboratory analysis may be helpful in differentiating the causes of fever. A full blood, including a platelet count, is nowadays available in many places and may replace the tourniquet test. Alternatively, there are many rapid bed site tests available now for the serological diagnosis of dengue [18]. Although the sensitivity of these tests is limited, they may still improve a clinical diagnosis that is imprecise. Especially the development of a rapid dengue antigen test is promising because it does not suffer from existing antibodies [19].

It is beyond the scope of this article to discuss the laboratory diagnosis of dengue at length; however, our observation of the large impact of dengue on the primary health services calls for further studies into the (cost-) effectiveness of tools that assist in diagnosing dengue. Since extra costs by introducing blood tests may, at least partially, be recovered by a reduced prescription of drugs, including antibiotics, such studies will also relevance for resource poor settings.

## Conclusion

In Binh Thuan province, southern Vietnam, dengue is one of the major causes of undifferentiated fever. It presents as a highly unspecific illness and is hardly recognized as a clinical entity by primary health care physicians. The results of this study support further studies on applying intervention measures to improve the diagnostic accuracy and precision at the primary healthcare level in dengue endemic regions.

## Competing interests

There is no conflict of interest.

## Authors' contributions

HLP: Fieldwork and data collection, data management, analysis and statistics, writing manuscript.

PJdV: Study design, fieldwork and data collection, data management, analysis and statistics, writing manuscript.

TTTN: Fieldwork and data collection, laboratory tests, data management

PTG: Fieldwork and data collection, data management

LQH: Fieldwork and data collection, data management

TQB: Fieldwork and data collection, data management, logistics and local scientific supervision, review and corrections of manuscript.

NVN: Fieldwork and data collection, review and corrections of manuscript

NN: Study design, data analysis and statistics, review and corrections of manuscript

PAK: Study design, review and corrections of manuscript

## Pre-publication history

The pre-publication history for this paper can be accessed here:


